# Immunohistochemical Expression of P16 in Cervical Lesions: An Observational Study

**DOI:** 10.31729/jnma.v63i2091.9228

**Published:** 2025-11-30

**Authors:** Shaheen Naaz Ansari, Radhika Kunwar, Sapana Amatya Vaidya

**Affiliations:** 1 Department of Gynecology and Obstetric, Paropakar Maternity and Women’s Hospital, Kathmandu, Nepal

**Keywords:** *cervical cancer*, *dysplasia*, *human papillomavirus*, *immunohistochemistry*, *p16 tumor suppressor protein*

## Abstract

**Introduction:**

Cervical cancer is the leading cancer and the predominant cause of cancer-associated deaths among women worldwide. Human papillomavirus infection is the foremost cause of cervical cancer. P16 tumor suppressor protein is overexpressed in high-risk HPV infected cells. This has led to the development of P16 as a reliable predictive biomarker to identify women with cervical dysplasia who are at risk of progressing to high-grade cervical intraepithelial neoplasia and invasive cancer. The study aimed to determine the expression pattern of P16 in lesions of the cervix.

**Methods:**

This observational study was conducted at Paropakar Maternity and Women’s Hospital, Nepal, from June 2025 to August 2025. All patients who visited the Gynecology outpatient department for abnormal cervical screening were included in the study. An immunohistochemistry test for P16 was performed in histologically confirmed pre-invasive and invasive lesions.

**Results:**

Of 88 cervical biopsies, 34 (38.63%) lesions were preinvasive, and 20 (22.72%) lesions were invasive. P16 immunohistochemistry was performed in 52 (59.09%) histologically confirmed dysplastic and invasive cervical lesions. Among 52 dysplastic lesions, 37 (71.15%) cases were P16-positive. Out of 37 P16-positive cases, 17 (45.95%) were preinvasive and 20 (54.05%) were invasive lesions. In pre-invasive group, 1 (10 %) were CIN1 lesion, 2 (28.57%) CIN II lesions, and 14 (93.33%) CIN III lesions were P16 positive, and among 20 invasive lesions, 20 (100%) were P16 positive.

**Conclusions:**

P16 expression was found to increase with the severity of cervical dysplasia and was positive in all invasive cervical cancer cases.

## INTRODUCTION

Cervical cancer (CC) is the most common cancer among women in Nepal.^1,2^ Human papillomavirus (HPV) is the cause of CC in 99.7% of cases.^3^ Majority of HPV infections are transient, while persistent high-risk HPV infections have the potential to progress to higher-grade cervical intraepithelial neoplasia (CIN) and CC. The overexpression of the tumor suppressor protein, P16, occurs as a defense against malignant transformation.

Histopathologic evaluation alone faces challenges due to interobserver variation and difficulty in predicting which cervical lesions might progress to carcinoma. Studies have shown that the immune-expression of P16 protein correlates with the degree of CIN and CC.^4-6^ Hence addition of P16 immunohistochemistry can predict the progression of dysplasia.

In this study, the P16 immunohistochemical expression in preinvasive and invasive cervical lesions was determined. This is the first descriptive study in Nepal to report P16 immuno-expression in cervical lesions, providing novel evidence to the existing body of scientific literature.

## METHODOLOGY

This observational study was conducted in the Gynecology outpatient department (OPD) at Paropakar Maternity and Women’s Hospital, Nepal, for a period of three months from June 2025 to August 2025. Following approval from the Institutional Review Board (IRB) of the National Academy of Medical Sciences (NAMS) (Reference no. 2612022183).

The census sampling method was applied. Women of all age groups attending the gynecology outpatient department (OPD) and those referred to the gynecology OPD for abnormal cervical screening who met the inclusion and exclusion criteria were enrolled in the study.

The inclusion criteria were: i) Abnormal cytology, ii) visual inspection with acetic acid (VIA) positive referrals from other centers, iii) HR HPV detected, iv) Direct tissue biopsy from clinically suspicious cervix and gross cervical lesion. The exclusion criteria were: i) Inadequate cervical tissue for histologic diagnosis, ii) Those currently undergoing treatment for cervical lesions, iii) Patients who did not give consent, iv) Ulcerated and necrotic tissue, and v) Critically ill patients.

Baseline demographic information and per speculum examination finding was recorded when a biopsy was decided. Women who had symptoms but a non-suspicious cervix were screened for cervical cancer, and the results were recorded. For women who were referred with abnormal cervical screening reports of either cytology or HPV DNA or both from other centers, their reports were reviewed. Based on the cervical screening report, colposcopy-guided cervical biopsy was performed according to hospital protocol. For women with a clinically suspicious cervix, colposcopy-guided biopsy was obtained irrespective of cytology or HPV screening. For those women who had visible growth in the cervix, cervical biopsy was directly obtained without colposcopy.

The specimen was sent to the Department of Pathology for Histopathological Examination (HPE) evaluation. The HPE report was categorized into benign lesions, preinvasive lesions, and invasive lesions. The preinvasive lesions were subcategorized into 3 grades of severity as per the Cervical Intraepithelial Neoplasia (CIN) classification system. The 3 grades of CIN lesions were CIN I lesion, CIN II lesion, and CIN III lesion. Invasive lesions were categorized into squamous cell carcinoma (SCC) and adenocarcinoma according to the histopathology. Following the HPE evaluation, with ethical approval from the IRB of NAMS, the dysplastic and malignant lesions were further processed for P16 immunohistochemistry to detect the presence of P16 protein, produced by the P16 tumor suppressor gene, in HPV-infected cells. Immunohistochemistry was done using a Mouse monoclonal antibody to p16, Clone JC2, IgG antibody.

Formalin-fixed tissue was dewaxed, rehydrated, and then antigen-retrieved. The slide was washed with buffer, and blocking was done using a peroxide block. The primary antibody was applied. This was followed by washing and the application of a secondary antibody. The P16 antibody binds to the P16 protein in the tissue. Then, washing and chromogen application were done, followed by washing and a counterstain application. Following this dehydration, clearing and mounting were done with distrene-plasticizer-xylene (DPX). Interpretation was done as follows: the presence of continuous and diffuse staining of both nuclear and cytoplasm of the cells in the basal and parabasal layer was reported as abnormal expression as per the Lower Anogenital Squamous Terminology Standardization (LAST) project guideline.^7^ In this study, the presence of abnormal expression of P16 was considered as P16-positive cases.

A cervical biopsy with IHC report was followed up on and recorded during the subsequent visit. Data were analyzed using IBM SPSS Statistics for Windows, Version 26.0 (IBM Corp., Armonk, NY, USA). Categorical variables were summarized using frequency and percentage, while numerical data were presented as mean±standard deviation.

## RESULTS

This study included 88 women out of which, 54 (61.36%) cases were dysplastic and invasive lesions in cervical biopsy. However, 2 (2.27%) cases of preinvasive lesions could not be included in the study as patients could not afford the test. Immunohistochemical of P16 staining was performed on 52 (59.09%) histologically confirmed dysplastic and invasive cervical lesions.

Among 88 cervical biopsies performed, 30 (34.09%) patients belonged to the 40-49 years of age group, 21 (23.86%) patients belonged to the 30-39 years of age group, and 19 (21.59%) patients belonged to the 50-59 years of age group. There were 60 (68.18%) patients who had married at < 20 years. 74 (84.09%) patients were multiparous. 55 (62.50%) patients had no history of tobacco use (Table 1).

**Table 1:** Baseline characteristics of study patients (n=88).

Indicators	n(%)
**Age (years)**	
20-29	8(9.09)
30-39	21(23.86)
40-49	30(34.09)
50-59	19(21.59)
60-69	6(6.81)
70-79	4(4.54)
**Age at Marriage (years)**	
<20	60(68.18)
≥20	28(31.81)
**Parity**	
Nulliparous	2(2.27)
Primiparous	12(13.63)
Multiparous	74(84.09)
**Tobacco Use**	
No	55(62.50)
Yes	33(37.50)

**Table 2:** P16 distribution pattern in cervical lesions.

Category	Positive	Negative
Pre-Invasive	17(53.12%)	15(46.87%)
CIN I	1(10.0%)	9(90.0%)
CIN II	2(28.57%)	5(71.42%)
CIN III	14(93.33%)	1(6.66%)
Invasive	20(100.0%)	0(0.0%)
Squamous cell carcinoma	19(100.0%)	0(0.0%)
Adenocarcinoma	1(100.0%)	0(0.0%)

**Figure 1:**
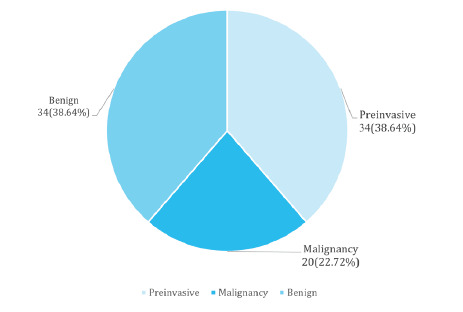
Histopathological diagnosis of cervical specimen (n=88).

Based on the histopathological diagnosis of 88 cervical biopsy specimens, 34 (38.64%) were benign lesions, 34 (38.64%) were preinvasive lesions, and 20 (22.72%) were invasive lesions (Figure 1). Among the 34 preinvasive lesions, 11 (32.35%) were diagnosed as CIN I, 7 (20.59%) as CIN II, and 16 (47.06%) as CIN III lesions. Malignant lesions were identified in 20 cases, of which 19 (95%) were squamous cell carcinoma and 1 (5%) was adenocarcinoma.

**Figure 2:**
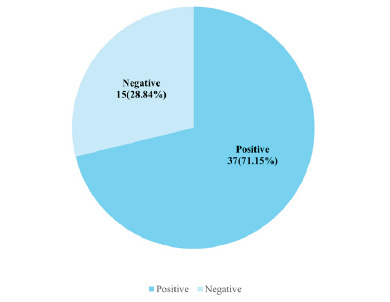
P16 Immunohistochemistry status in dysplastic and malignant lesions (n=52).

There were 54 histopathologically confirmed cases of dysplastic and invansive lesion. However, 2 patients could not afford the test so only 52 cases were sent for P16 Immuno-histochemistry test. Out of 52 histopathologically confirmed cases, 37 (71.15%) cases were P16 positive (Figure 2).

In the preinvasive group, 17 (53.12%) cases were positive for P16, while all 20 (100%) malignant cases were p16 positivite. In preinvasive group, there were 1 (10%) of CIN I, 7 (28.6%) of CIN II, 14 (93.3%) of CIN III lesions positive for P16. In invasive group, 20 (100%) malignant lesions were p16 positive.

## DISCUSSION

The overexpression of P16 is emerging as a promising biomarker to identify the process of transformation of HPV infected cervical lesions to cancer. The majority of the HPV infections regress without treatment, but 4% to 10% infected patients develop persistent HPV infections, leading to preinvasive and invasive cervical lesions.^8^ Tumor suppressor gene located on chromosome 9p21-22 produces P16 protein in defense against the oncogenic transformation of HPV infected cervical lesions.^9,10^

In the current study, the age of the patients affected by the lesions of the cervix was observed to be between 20 and 79 years, and the mean age of these affected women was found to be 40-49 years, with an increase in the proportion of SCC in 50-59 years. These finding aligns with the mean age of development of cervical cancer, which is known to be 47 years. Similar to our observation, the mean age of all cases of cervical lesion observed in the study of Pandey et al. was 48.28 ± 8.9 years.^11^ Evidences from several previous studies indicate that P16 expression is affected by age.^12^ In concordance with our observation, Krishnappa et al.^13^ also reported that the age of patients affected by cervicitis, preneoplastic lesions of the cervix, and cancer of the cervix fell between 21 and 85 years. In their study also the mean age of all cervical lesions was 49.2 years, while the mean age of cervical cancer was 58.9 years. Similar findings were observed in both their study and our study. The mean age of cervical cancer reported by Ssedyabane et al.^14^ was between 40-49 years, and the mean age of CIN was 30-39 years, which is different from our findings. Disease distribution in different age groups is directly linked with the P16 expression in these age groups.

In our study, out of 88 cervical biopsy specimens studied, 34(36.8%) cases had benign lesions, 34(36.8%) cases had premalignant while 20(22.72%) had malignant lesions. Among the 34 preinvasive lesions, 11 (32.35%) had CIN I, 7 (20.59%) had CIN II, 16(47.06%) had CIN III. The referrals to the oncology unit for higher suspicion of abnormal lesions explain this finding of more CIN III lesions in this. The finding of the highest proportion of CIN III is also demonstrated by the study of Zhang et al., with 18 cases of LSIL and 33 cases of HSIL. ^15^

On immunohistochemistry of the 52 dysplastic lesions, 37(71.15%) of all the lesions showed positive staining with P16, while 15(28.84%) were negative for P16. Prevalence of positive P16 lesions in previous studies has been found to be between 64.70% to 75% of dysplastic lesions.^14,16^ Our results show agreement with this prevalence rate.

Of the 32 preinvasive lesions tested for P16, 17(53.12%) stained positive for P16. In contrast to our finding higher rate of P16 positivity in preinvasive lesions has been reported in other studies. In the study of Krishnappa et al, P16-positive preinvasive lesions were reported to be 72%.^13^ A progressively increasing pattern of P16 positivity with increasing dysplasia was found among the 3 groups of preinvasive lesions in our study, which were 1(10%) in CIN I, 2(28.57%) in CIN II, and 14(93.33%) in CIN III lesions. The progressive increase in P16 expression from CIN I to CIN III matches with the study of Abdel-Hakam FA et al.^17^ Their study demonstrated positivity rate of 10.7% in CIN I lesion, 92.6% in CIN II lesion and 100.0%, in CIN III lesion, Similar increasing trend of P16 positivity from CIN I through CIN III is also seen in the study of Saigal et al.^18^ and Zhang et al.^15^ In the study of Hu et al., P16 positivity rate were 75.0%(LSIL), 96.3%(HSIL) and 100%(invasive cancer). ^19^ The study of Benevolo M et al.^20^ have reported similar progressively increasing expression of P16 with increasing dysplasia. They reported 31% CIN I, 90% CIN II, and 100% CIN III expression of P16 in their study. Studies have stated that increased cellular stress in higher grades of dysplasia increases the degree of expression of P16.^21^ The cellular stress in higher degrees of dysplasia is more than that of lower grades of dysplasia.

All 20(100%) of invasive lesions in our study were P16 positive similar to the finding of Hu et al.^19^ The uniform expression of P16 observed across all cases of invasive lesions including both squamous cell carcinoma and adenocarcinoma reflects that P16 is biomarker for both squamous and glandular lesion and also relates to the established etiological role of persistent high risk HPV infection of cervix with oncogenic transformation of pervasive lesions to invasive lesion.

The lower expression of P16 in CIN I and CIN II in our study could be due to low-risk HPV infection causing less inactivation by RB, hence lesser expression of P16 than the high-risk type HPV infection showing higher grades of dysplasia. The affinity of the E7 protein for the Rb of low-risk HPV is much lower than that of high-risk HPV types. Due to this, P16 staining in low-risk HPV appears as weak and focal staining of nuclei and cytoplasm in the intermediate and superficial layers only.

Despite these associations application of P16 as a screening tool for precancerous cervical lesions needs specialized laboratory and personnel and increased cost, which can be a limiting factor for its application in low-income countries. Limitations of the study were a short period of study and a lack of P16 study in benign lesions.

## CONCLUSION

P16 expression increased with the severity of cervical lesions and was present in all cases of invasive cervical cancer. The study showed a gradual rise in P16 positivity from low-grade to high-grade lesions.

## Data Availability

The data are available from the corresponding author upon reasonable request.
